# Efficacy of different spinal cord stimulation paradigms for the treatment of chronic neuropathic pain (*PARS*-trial): study protocol for a double-blinded, randomized, and placebo-controlled crossover trial

**DOI:** 10.1186/s13063-020-05013-7

**Published:** 2021-01-25

**Authors:** Rezvan Ahmadi, Benito Campos, Mohammad Mehdi Hajiabadi, Colette Doerr-Harim, Solveig Tenckhoff, Dirk Rasche, Andreas Unterberg, Jan Vesper, Tom Bruckner, Volker Tronnier

**Affiliations:** 1 Department of Neurosurgery, University Hospital Heidelberg, INF 400, 69120 Heidelberg, Germany; 2 The Study Center of the German Surgical Society (SDGC), University Hospital Heidelberg, Heidelberg, Germany; 3 Department of Neurosurgery, University of Lübeck, Lübeck, Germany; 4 Department of Functional Neurosurgery and Stereotaxy, Heinrich-Heine-Universität Düsseldorf, Düsseldorf, Germany; 5 Institute of Medical Bioinformatics, University of Heidelberg, Heidelberg, Germany

**Keywords:** Spinal cord stimulation, Neuropathic pain, Wireless stimulation, Randomized controlled trial

## Abstract

**Background:**

Spinal cord stimulation (SCS) is an effective method to treat neuropathic pain; however, it is challenging to compare different stimulation modalities in an individual patient, and thus, it is largely unknown which of the many available SCS modalities is most effective. Specifically, electrodes leading out through the skin would have to be consecutively connected to different, incompatible SCS devices and be tested over a time period of several weeks or even months. The risk of wound infections for such a study would be unacceptably high and blinding of the trial difficult. The PARS-trial seizes the capacity of a new type of wireless SCS device, which enables a blinded and systematic intra-patient comparison of different SCS modalities over extended time periods and without increasing wound infection rates.

**Methods:**

The PARS-trial is designed as a double-blinded, randomized, and placebo-controlled multi-center crossover study. It will compare the clinical effectiveness of the three most relevant SCS paradigms in individual patients. The trial will recruit 60 patients suffering from intractable neuropathic pain of the lower extremities, who have been considered for SCS therapy and were already implanted with a wireless SCS device prior to study participation. Over a time period of 35 days, patients will be treated consecutively with three different SCS paradigms (“burst,” “1 kHz,” and “1.499 kHz”) and placebo stimulation. Each SCS paradigm will be applied for 5 days with a washout period of 70 h between stimulation cycles. The primary endpoint of the study is the level of pain self-assessment on the visual analogue scale after 5 days of SCS.

Secondary, exploratory endpoints include self-assessment of pain quality (as determined by painDETECT questionnaire), quality of life (as determined by Quality of Life EQ-5D-5L questionnaire), anxiety perception (as determined by the Hospital Anxiety and Depression Scale), and physical restriction (as determined by the Oswestry Disability Index).

**Discussion:**

Combining paresthesia-free SCS modalities with wireless SCS offers a unique opportunity for a blinded and systematic comparison of different SCS modalities in individual patients. This trial will advance our understanding of the clinical effectiveness of the most relevant SCS paradigms.

**Trial registration:**

German Clinical Trials Register, DRKS00018929. Registered on 14 January 2020.

**Supplementary Information:**

The online version contains supplementary material available at 10.1186/s13063-020-05013-7.

## Background

Spinal cord stimulation (SCS) is an effective method to treat neuropathic pain of the lower extremity. A typical SCS device transmits electric current to the posterior columns of the spinal cord through a thin, epidural electrode. This electric current interferes with physiological pain transmission; however, the exact mechanisms of pain suppression are not fully understood. The epidural electrode is wired to an extension cable leading out through the patient’s skin, where it is connected to an implantable pulse generator (IPG) during SCS testing. To minimize the risk of local wound infection and transmission of wound infections to the epidural space, testing is usually constrained to a few days. If a patient confirms substantial pain relief, the extension wire and the IPG are implanted subcutaneously in a second surgery and can be operated by the patient via remote control.

SCS has been used in the clinic for five decades; however, it is largely unknown which of the many available SCS modalities is most effective [1]. This is mainly due to the fact that it is extremely challenging to compare all the different stimulation modalities in an individual patient and during a short test phase. On the one hand, SCS devices are manufactured by different providers, are limited to certain proprietary SCS modalities, and are largely incompatible with other SCS devices. Even in the absence of compatibility issues, electrodes leading out through the skin would have to be consecutively connected to different SCS devices and be tested over a time period of several weeks/months. The risk of wound infection for such a study would be unacceptably high and blinding of the trial difficult. Several studies have compared different SCS paradigms, but these SCS modalities were limited to those available for the particular SCS device employed. For instance, Demartini et al. and De Ridder et al. compared burst and tonic stimulation [2, 3]. Duse et al., on the other hand, studied the clinical effectiveness of burst and 1 kHz SCS. Finally, other studies compared 10 kHz stimulation with traditional paresthesia-inducing SCS [4, 5] or different frequencies of SCS (1, 4, 7, and 10 kHz SCS) using the same stimulation device [6].

The PARS-trial seizes the capacity of a new type of wireless SCS device (Stimwave, Florida, USA), which was approved for SCS in recent years [7]. Consisting of an electrode with an integrated microchip and a separate miniature receiver, this setting does not require transcutaneous electrodes and can be implanted in a single, minimally invasive surgical procedure. Most importantly, SCS testing can be extended ad libitum without increasing wound infection rates.

This technological advance coincides with a second development, namely the development of paresthesia-free SCS modalities [1]. These SCS paradigms do not cause paresthesia and, in principle, cannot be distinguished by patients. Three of the most prevalent SCS paradigms are paresthesia-free: high-density stimulation (1 kHz), high-frequency stimulation at > 1 kHz, and burst stimulation (burst) [1]. High-frequency stimulation and burst are two forms of SCS specifically developed to suppress lower back pain. High density, on the other hand, stimulates at 1 kHz with high pulse width and low amplitude to produce an almost permanent suppression of neuronal activity.

Combining paresthesia-free SCS modalities with wireless SCS offers a unique opportunity for a blinded and systematic comparison of different SCS modalities in individual patients.

## Methods/design

### Aim of the study

The study aims to compare the clinical effectiveness of four SCS modalities in individual patients. SCS modalities include three prevalent paresthesia-free modalities (“burst,” “1 kHz,” and “1.499 kHz”) and placebo stimulation.

### Study design

This trial is a prospective multi-center, double-blinded, randomized, and placebo-controlled crossover trial.

### Trial sites

The PARS-trial will recruit patients at a total of 10 centers, including university hospitals and regional hospitals located throughout Germany. Given that participating centers estimated the numbers of enrolled patients to be roughly 2–3 patients/year, we opted for a higher number of hospitals to complete the study in approximately 2 years.

### Patient inclusion criteria

The trial will recruit patients suffering from intractable neuropathic pain, who have been considered for SCS therapy according to the German guidelines and, prior to the study, were already implanted with a wireless SCS device (Stimwave, Florida, USA). Inclusion criteria require placement of a single epidural electrode with the distal tip located between the 8th and the 12th thoracic vertebra. Patients with pain in the lower extremity are eligible for this trial. Pain distribution can be unilateral or bilateral. Patients with concomitant lower back pain are eligible too, as long as pain in the lower extremities predominates. Duration of pain history should be longer than 6 months and shorter than 5 years. This choice was based on a study by Kumar et al. [8] which assessed the impact of wait times on spinal cord stimulation therapy outcomes. The authors came to the conclusion that best outcomes were achieved within the first 5 years after symptom onset. In addition, our trial only considers patients who have been found eligible for SCS therapy according to the German guidelines and with pain symptoms persisting for at least 6 months.

The trial will exclusively include adults (≥ 18 years of age) who provide written informed consent after (a) receiving detailed information pertaining to the trial (including a detailed patient information brochure which was approved by the local ethics committee), (b) having at least 24 h to consider trial participation, and (c) having no further questions at the time of consent. Only dedicated study physicians who have been trained and authorized by the principal investigator will recruit patients and/or gain informed consent. None of the physicians involved in the initial treatment, i.e., implantation of the wireless SCS device, will be involved in recruiting patients and/or gaining informed consent. Patients will be approached after implantation of a wireless SCS device by study physicians who will explain the study in detail. Should the patient consider study participation, he or she will be handed a detailed information sheet which has been reviewed and approved by the local IRB. All patients must be able to understand the nature and the extent of the trial.

### Patient exclusion criteria

Exclusion criteria include coverage of pain area of less than 90% during intraoperative testing (using paresthesia-inducing stimulation). Patients suffering from other pain syndromes such as ischemic pain (e.g., peripheral artery disease) and primary chronic pain will not be included in the trial. Exclusion criteria further include coagulopathy, pregnancy, neurodegenerative disease, lack of understanding of the trial, and any planned changes in existing pain medication for the duration of trial participation. We opted against exclusion criteria based on age limit but rather included neurodegenerative disease and lack of understanding of the trial as exclusion criteria to better reflect the biological age of patients.

### Patient withdrawal criteria

Patients can revoke their consent to participate in the trial at any time, in writing or verbally and without giving reasons for their decision. Patients who revoke their consent can decide whether their trial data should be included in the final analysis. Individual termination criteria with regard to trial participation include withdrawal of the patient’s consent to participate in the trial, severe wound infection leading to explantation of the SCS device, and any type of morbidity or patient discomfort leading to explantation of the stimulator.

### Assignment of intervention, randomization, and blinding

The study is planned as a fourfold crossover study using a Williams design. In a crossover design, patients are not randomly assigned to treatments, but rather treatment sequences are randomized. Such a crossover design avoids inter-patient variability and reduces the required sample size to power this trial. On the other hand, it increases the length of the intervention for each individual patient and thus, ultimately, might negatively influence patient compliance.

Since some part of the SCS effect observed at early stages of therapy may be the result of a placebo effect, and some studies have reported placebo effects comparable to SCS effects [9], the study also includes a placebo control, i.e., a time interval in which there is no stimulation and which should be indistinguishable from paresthesia-free SCS.

Each patient is treated with four SCS modalities (“burst,” “1 kHz,” “1.499 kHz,” and “placebo”). All stimulation sequences are generated at the beginning of the trial, and each patient will be randomly assigned to one of 24 unique treatment sequences. An independent technician will program the SCS modalities as well as the order of the different stimulation modalities into the wireless SCS control hardware prior to the intervention.

A randomization list was generated prior to trial initiation. The designated trial statistician had no access to the lists and was not involved in the generation of the randomization list. A randomization number (1–500) was randomly assigned to one of the 24 unique treatment sequences (e.g., randomization #1: “1 kHz”-“placebo”-“1.499 kHz”-“burst,” randomization #2: “burst”-“placebo”-“1 kHz”-“1.499 kHz”). This list was further divided into blocks of equal length so that each participating center would receive a list of randomized sequences and a matching number of sealed envelopes containing one treatment sequence per envelope (e.g., center #1 would receive randomization numbers and the corresponding treatment sequences 1–50 as well as 50 sealed envelopes with treatment sequences 1–50, center #2 would receive randomization numbers and the corresponding treatment sequences 51–100, as well as 50 sealed envelopes with treatment sequences 51–100).

The following procedure is applied for allocation: patients are screened and enrolled at each center. Study personnel will remove sealed randomization envelopes from the investigator site file. Sealed randomization envelopes are then handed out to an independent technician who opens the randomization envelope and uses the treatment sequence to program the wireless SCS device.

Study participants and physicians will be blinded to the order of the four stimulation modalities. The four different stimulation modalities will only be known to these two parties as programs 1–4. In coordination with the study center, patients will use the remote control of their SCS device to select the pre-set programs in consecutive order. There will be no contact with the independent technician for the duration of trial participation (35 days), and study personal must confirm double-blinding at the end of the study period. An emergency plan to unblind the treatment sequence was installed and can be enacted, e.g., in case the treatment sequence is required to replace a SCS device.

### Methods to reduce other types of bias

All centers participating in the study will be proficient in the implantation of the wireless SCS device, i.e., have more than 1 year of experience implanting the device and/or have attended an implantation course offered by the Department of Neurosurgery at Heidelberg University Hospital. In addition, existing pain medication will remain unchanged for the duration of trial participation unless the patient’s condition requires changes in pain medication. Potential changes in pain medication will be recorded. Study participants are instructed to record each day, if there were any changes in medication. Any change of study medication within the intervention period will lead to study withdrawal.

### Intervention

Seven days after implantation of the wireless SCS device, patients are contacted by the respective study center and asked to quantify pain on the visual analogue scale (VAS) and to complete the study questionnaires (Table 1). Patients are then requested to activate the first SCS modality which is known to them only as program 1. One hundred twenty hours after activation of the first SCS modality, patients are contacted once more by the respective study center and asked to quantify pain on the VAS and complete the study questionnaires. Patients are then requested to switch off SCS. After a washout period of 70 h, patients are contacted again by the respective study center. Once more, patients are asked to quantify pain on the VAS and complete the study questionnaires. Patients are then requested to activate the second stimulation program which is known to them only as program 2. This procedure (stimulation, test battery, washout phase, test battery, activation of the next SCS modality) is repeated until patients have been consecutively treated with all four SCS modalities.

**Table 1:**
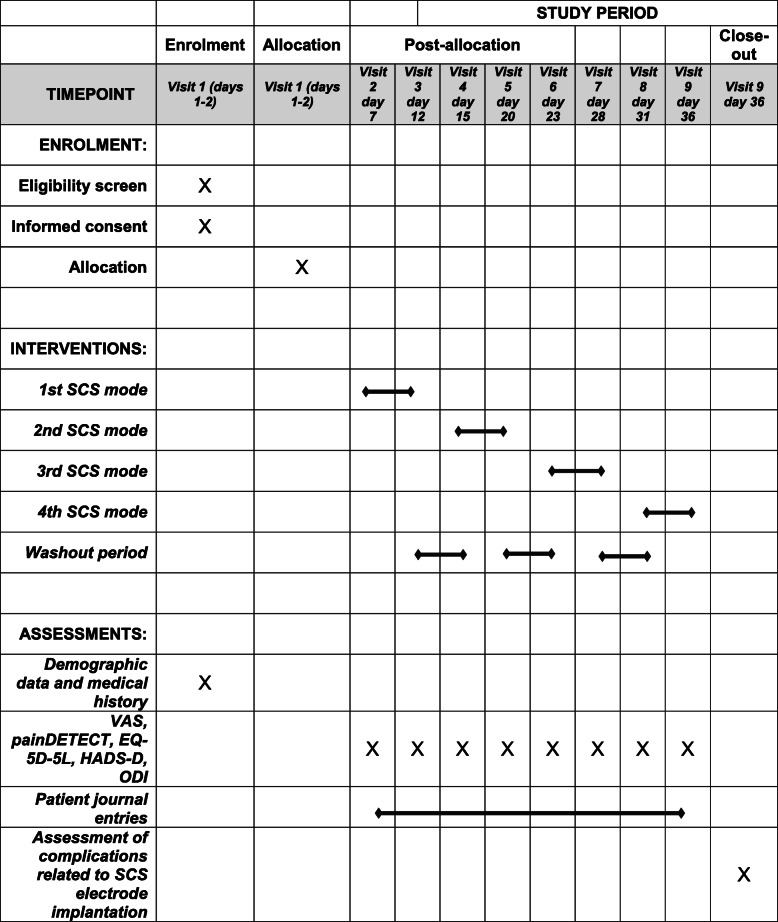
Schedule of enrolment, interventions, and assessments

### Primary endpoint

Primary endpoint is the level of pain measured on the visual analogue scale (VAS) after 120 h of SCS. VAS is a globally used instrument where patients rank their pain on a scale from 0 to 100. “0” corresponds to the pain-free state and “100” to the maximum imaginable state of pain. VAS is the most prevalent endpoint for studies involving SCS. In addition to VAS rating, patients are asked to assess which SCS program was most beneficial to them during their final visit at day 36.

### Secondary endpoints

Secondary endpoints include self-assessment through four validated questionnaires: pain quality as determined by painDETECT questionnaire (painDETECT), quality of life as determined by Quality of Life EQ-5D-5L questionnaire (EQ-5D-5L), anxiety perception as determined by the German version of the Hospital Anxiety and Depression Scale (HADS-D), and physical restriction as determined by the Oswestry Disability Index (ODI). All questionnaires are standardized and well established in SCS research [10–13].

In addition, demographic and study-related data are collected by the respective study centers during the screening visit (immediately after consent/inclusion of study participants) and during the final visit, which coincides with the regular clinical follow-up on day 36 of the trial. Data include electrode position (position of the distal electrode tip), medical history, potential complications related to SCS implantation, and total hours of stimulation per day.

### Patient timeline and trial visits

Routine implantation of the wireless SCS device occurs prior to the trial and as part of regular patient care (day 0). On days 1 and 2, patients are informed about the trial, and upon consent, inclusion and exclusion criteria are reviewed. Provided that all inclusion criteria and no exclusion criteria are met, an independent technician programs the previously randomized order of the four different SCS modes (“burst,” “1 kHz,” “1.499 kHz,” and “placebo”) into the SCS hardware. To patients, these four SCS modes are only known as programs 1, 2, 3, and 4. All patients are thoroughly instructed in the general handling of the SCS device. For a total of 7 days after implantation, SCS is switched off to wash out any potential effect caused by electrode placement during the preceding SCS surgery.

After this first washout phase (day 7) and with SCS switched off, patients are contacted by the respective study center. Patients are asked to assess pain on the VAS and are requested to fill out four questionnaires (painDETECT, EQ-5D-5L, HADS-D, and ODI). Time needed to complete the questionnaires is estimated at 1 h. Patients are instructed to activate the first stimulation mode immediately after completing the questionnaires. Specifically, patients are instructed to use each stimulation mode for 120 h and to stimulate at least 8 h per day. In addition, patients are asked to record daily stimulation durations in a patient journal, i.e., since it is not possible to record stimulation times with the wireless SCS device directly, patients are handed a diary after enrolment. This diary contains one page for every single day of the intervention. Study participants are instructed to record each time during the day when SCS was switched on and when SCS was switched off. If there are multiple periods of SCS during a day, patients are instructed to record stimulation times for each SCS period.

After 120 h of stimulation with the first stimulation mode (day 12), patients are contacted by the respective study center and asked to assess pain on the VAS and to fill out the four questionnaires (painDETECT, EQ-5D-5L, HADS-D, and ODI). After completing the questionnaires, patients are instructed to switch off stimulation. Now follows a washout phase of 70 h, during which stimulation remains switched off.

After the washout phase (day 15) and with stimulation still switched off, patients are contacted by the respective study center and asked to assess pain on the VAS and to fill out the four questionnaires (painDETECT, EQ-5D-5L, HADS-D, and ODI). Patients are then instructed to activate the second stimulation mode immediately after completing the questionnaires. This procedure (stimulation for 120 h, questionnaires, 70 h of washout phase, questionnaires, activation of the next SCS modality) is repeated exactly until the patient has been subjected to all four SCS modalities.

On day 36 (after 120 h of stimulation with the fourth stimulation modality), patients present for an outpatient appointment for regular clinical follow-up at the respective trial site, which coincides with the last study visit. Patients are asked once more to assess pain on the VAS and to fill out the four questionnaires (painDETECT, EQ-5D-5L, HADS-D, and ODI) and are asked to assess which SCS program was most beneficial to them overall. An independent technician performs a final paresthesia-inducing test stimulation to confirm that the pain area is still covered by at least 90%. The patient then chooses the preferred stimulation mode.

### Data management

Data management is carried out according to the Standard Operating Procedures (SOPs) of the Institute for Medical Biometry and Informatics (IMBI) Heidelberg. All demographic and trial-related data collected within the scope of this trial are entered by the respective trial sites into an electronic Case Report Form (eCRF) implemented in the Research Electronic Data Capture (REDCap™) system (www.project-redcap.org). Completeness, validity, and plausibility of the data are checked by a data validation program. Questionnaires filled out by patients in paper form as part of the trial are forwarded by trial sites to the IMBI for data entry. Double data entry will be performed where applicable.

### Sample size calculation

Sample size calculations are based on a previous SCS study protocol by Kriek et al. [14]. Analogous to this study protocol, we chose a minimal detectable effect size of 0.15 (i.e., 15%) on our primary outcome parameter, arriving at a required sample size of 48 patients with a power of 82.9% and a significance level of 0.05. Due to the statistical design of our trial, it will not be possible to censor or replace study participants after study termination. For this reason, a termination/dropout rate of 20% was assumed for sample size calculations and the total number of patients increased from *n* = 48 to *n* = 60. In the case of a drop in the number of trial participants below the minimum number of *n* = 48, the study protocol provides for the replacement of these participants by the recruitment and inclusion of additional study participants. To reduce the likelihood of missing data, four measures will be implemented: (1) Patients will be sensitized by study personnel and on several occasions to the fact that adherence to the study protocol is indispensable for study success. Date and time of each prospective study visit are explained to study participants and entered into a calendar which patients receive immediately after enrolment. (2) Study participants will be contacted via phone by study centers each time they have to assess stimulation effects and have to fill out questionnaires. During these phone calls, patients will be sensitized again to the fact that missing data might compromise the study. If a study participant cannot be reached within an agreed period of time, he or she will be made aware again during the first subsequent contact that a complete and precise procedure is essential for the success of the individual study. (3) A data validation plan was implemented. This plan generates automatic and instantaneous queries if data entry by study personnel is incomplete or implausible (e.g., date of intervention predates date of enrolment). (4) During the last study visit, study personnel screens each patient’s questionnaires to identify missing data. If missing patient data is identified and retrospective data collection does not compromise study results, patients are asked to provide missing data (e.g., patients will be asked to provide a missing name, a missing date, or a missing medication name but will not be asked to “remember” how much pain relief they had during a particular stimulation cycle). Sample size calculation was done using PASS Vs. 16.03 and was based on previous work by Chow et al. [15].

### Analysis variables and statistical methods

All variables will be reported by tabulation of the measures of the empirical distributions. According to the scale levels of the variables, means, standard deviations, medians, and minimum and maximum or absolute and relative frequencies will be reported. Descriptive *p* values of the corresponding statistical tests (*t* test in the case of continuous data, chi-square test for categorical data) and confidence intervals will be reported. The primary endpoint, pain perception on the VAS, will be evaluated using a linear mixed model with sequence, period, and treatment as fixed factors and patient as random factor. Statistical analysis will be done with SAS Vs. 9.4 or higher and the procedure *proc mixed*.

### Study-related risks

Since implantation of the wireless SCS device takes place independently and prior to study participation, rates of wound infection and technical failure are not affected by study participation.

### Clinical data monitoring

Monitoring is carried out by the Study Center of the German Surgical Society (SDGC) according to the SDGC’s SOPs and in compliance with the ICH-GCP guideline (E6). Monitoring focuses on patient safety and patient rights, on compliance with the study protocol as well as on data validity. All investigators must provide access to the SDGC’s independent monitors to study specific data and allow on-site visits before, during, and after study completion. Investigator site files are created and made available to the centers.

## Discussion

The PARS-trial will compare the clinical effectiveness of the three most relevant SCS paradigms in individual patients. To our knowledge, the wireless SCS device employed in this study is the only device that can reproduce all relevant paresthesia-inducing and paresthesia-free SCS modalities. Our crossover study design seizes the advantage of wireless SCS, namely the possibility to test several different SCS modalities in one and the same patient, thus avoiding inter-patient variability and reducing the required sample size to power this trial.

But even with this wireless SCS technology, it will be extremely difficult to compare paresthesia-inducing and paresthesia-free SCS modalities in a blinded fashion. The PARS-trial is thus restricted to paresthesia-free SCS modalities. In addition, patients are required to recharge their SCS device every 6 h, preventing patients from drawing conclusions about individual stimulation modalities based on different battery runtimes.

To our knowledge, there is not enough systematic data to assess the optimal washout period. A recent review by Duarte et al. [9] identified 12 RCTs of SCS. Washout periods were heterogeneous: 4 RCTs did not include a washout period between SCS modalities. In studies that included a washout period, periods included 15 min, 12 h, 2 days, and 1 week. Given such uncertainty regarding the proper length of the washout period, we considered a 70-h washout period to be a good trade-off between a conservative approach and an appropriate length of the intervention period. A washout period lasting 70 h instead of 72 h was chosen to avoid time shifts during the intervention period. Time required to fill out questionnaires was estimated at 1 h. Since one round of questionnaires would be filled out after 5 days of SCS and one round of questionnaires after the subsequent washout period, the next SCS cycle would start 72 h + 2 h later in the alternative scenario, i.e., each subsequent SCS cycle would start 2 h later during the day (e.g., first SCS cycle starts at 4 pm, second SCS cycle starts at 6 pm, and third SCS cycle has to start at 8 pm at night). To keep times consistent for trial participants and to make it easier for them to integrate SCS times into their daily routines, we reduced the washout period to 70 h. Accordingly, and from a trial participant’s perspective, SCS and washout periods would thus always start at the same time during the day.

Since the wireless SCS device does not record stimulation duration, the PARS study protocol will rely on patients’ compliance to stimulate an average of 8 h a day and record stimulation times in a patient’s diary. An average of 8 h per day was chosen based on our previous experience with the wireless SCS system. We recently evaluated patients suffering from therapy-resistant neuropathic pain, who were implanted with a wireless SCS system at our institution [16]. Patients reported that longer stimulation periods were difficult to integrate in their daily (working) routines and very few of them required SCS at night.

From these results and from current practice, we also concluded that many patients who benefited from wireless SCS already benefited within 72 h, even though, in several cases, the full SCS benefit only materialized after several weeks. While extending each stimulation period in the abovementioned trial from 5 days to several weeks would be technically feasible, we would also anticipate a strong and negative effect on patient compliance. Altogether, we are convinced that 5 days of SCS per stimulation paradigm is the best trade-off between response times experienced in our pilot study and an appropriate length of the intervention period.

In our experience, optimal electrode position (i.e., placement of the distal electrode tip) ranges from Th8 to Th12 for most patients. Thus, this range reflects a compromise between the need for a standardized electrode localization and the need to adapt epidural position to individual patients in order to achieve high (≥ 90%) pain coverage.

An important feature in our study protocol is the washout period between each stimulation modality. There is little knowledge regarding optimal washout periods after initial SCS implantation and after SCS treatment. For our study protocol, we have thus chosen a conservative approach, i.e., 7 days of washout period after initial implantation and 3 days after each SCS cycle.

Finally, during a recent evaluation of the wireless SCS system which was conducted at our institution [16], we had the opportunity to assess questionnaire fatigue in our patients. Based on this pilot experience, it seemed feasible to conduct repeated surveys over a 35-day intervention period.

### Trial status

This manuscript is based on the current version of the study protocol (version 3.0, last updated on September 30, 2020). Recruitment of patients for the trial has started in April 2020. The clinical phase of the trial (last patient out) is expected to be completed in December 2022.

## Supplementary Information

**Additional file 1.** SPIRIT 2013 Checklist: Recommended items to address in a clinical trial protocol and related documents.

## Data Availability

Not applicable, no datasets are included in this study protocol.

## Abbreviations

SCS: Spinal cord stimulation
VAS: Visual analogue scale
CRF: Case Report Form
DRKS: German Clinical Trials Register
GCP: Good Clinical Practice
ICH: International Conference on Harmonization of Technical Requirements for Registration of Pharmaceuticals for Human Use
IMBI: Institute of Medical Bioinformatics
QoL: Quality of life
SDGC: Study Center of the German Surgical Society
painDETECT: painDETECT questionnaire
EQ-5D-5L: Quality of Life EQ-5D-5L questionnaire
HADS-D: German version of the Hospital Anxiety and Depression Scale
ODI: Oswestry Disability Index
SOPs: Standard Operating Procedures
