# Supplementary material for: Efficacy of different spinal cord stimulation paradigms for the treatment of chronic neuropathic pain (PARS-trial): study protocol for a double-blinded, randomized, and placebo-controlled crossover trial
Source: Trials. 2021 Jan 25;22:87. doi: 10.1186/s13063-020-05013-7 (PMC7830748; doi:10.1186/s13063-020-05013-7)
Supplement: Supplementary file 1 — Additional file 1. SPIRIT 2013 Checklist: Recommended items to address in a clinical trial protocol and related documents. [file 13063_2020_5013_MOESM1_ESM.doc]

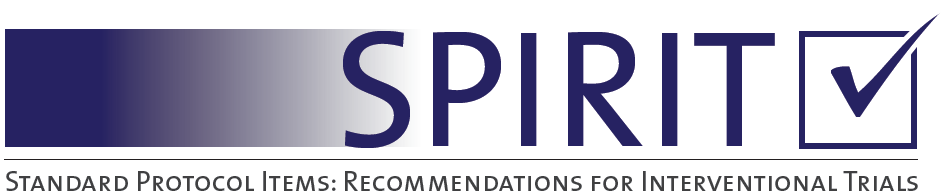


SPIRIT 2013 Checklist: Recommended items to address in a clinical trial protocol and related documents*

| Section/item | ItemNo | Description |
| --- | --- | --- |
| **Administrative information** | | |
| Title | 1 | Descriptive title identifying the study design, population, interventions, and, if applicable, trial acronym (addressed on page #1) |
| Trial registration | 2a | Trial identifier and registry name. If not yet registered, name of intended registry (addressed on page #3) |
|  | 2b | All items from the World Health Organization Trial Registration Data Set (NA, all items accessible through url of primary registry – 2a) |
| Protocol version | 3 | Date and version identifier (addressed on page #15-16) |
| Funding | 4 | Sources and types of financial, material, and other support (addressed on page #16) |
| Roles and responsibilities | 5a | Names, affiliations, and roles of protocol contributors (addressed on page #1, 16, 17) |
|  | 5b | Name and contact information for the trial sponsor (addressed on page #1, 16) |
|  | 5c | Role of study sponsor and funders, if any, in study design; collection, management, analysis, and interpretation of data; writing of the report; and the decision to submit the report for publication, including whether they will have ultimate authority over any of these activities (addressed on page #16) |
|  | 5d | Composition, roles, and responsibilities of the coordinating centre, steering committee, endpoint adjudication committee, data management team, and other individuals or groups overseeing the trial, if applicable (see Item 21a for data monitoring committee) (addressed on page #1, 12, 13) |
| Introduction |  |  |
| Background and rationale | 6a | Description of research question and justification for undertaking the trial, including summary of relevant studies (published and unpublished) examining benefits and harms for each intervention (addressed on page #4,5,6) |
|  | 6b | Explanation for choice of comparators (Placebo-controlled trial: Comparing paresthesia-free SCS modalities allows for a blinded and systematic comparison of different SCS modalities in individual patients. Since some part of the SCS effect observed at early stages of therapy may be the result of a placebo effect, and some studies have reported placebo effects comparable to SCS effects, this study includes a placebo stimulation, i.e. a time interval in which there is no stimulation and which should be indistinguishable from paresthesia-free SCS.) |
| Objectives | 7 | Specific objectives or hypotheses (addressed on page #8) |
| Trial design | 8 | Description of trial design including type of trial (eg, parallel group, crossover, factorial, single group), allocation ratio, and framework (eg, superiority, equivalence, noninferiority, exploratory) (addressed on page #6) |
| Methods: Participants, interventions, and outcomes | | |
| Study setting | 9 | Description of study settings (eg, community clinic, academic hospital) and list of countries where data will be collected. Reference to where list of study sites can be obtained (addressed on page #6) |
| Eligibility criteria | 10 | Inclusion and exclusion criteria for participants. If applicable, eligibility criteria for study centres and individuals who will perform the interventions (eg, surgeons, psychotherapists) (addressed on page #6,7) |
| Interventions | 11a | Interventions for each group with sufficient detail to allow replication, including how and when they will be administered (addressed on page #9) |
|  | 11b | Criteria for discontinuing or modifying allocated interventions for a given trial participant (eg, drug dose change in response to harms, participant request, or improving/worsening disease) (addressed on page #7,13) |
|  | 11c | Strategies to improve adherence to intervention protocols, and any procedures for monitoring adherence (eg, drug tablet return, laboratory tests) (addressed on page #10,11,13) |
|  | 11d | Relevant concomitant care and interventions that are permitted or prohibited during the trial (addressed on page #8) |
| Outcomes | 12 | Primary, secondary, and other outcomes, including the specific measurement variable (eg, systolic blood pressure), analysis metric (eg, change from baseline, final value, time to event), method of aggregation (eg, median, proportion), and time point for each outcome. Explanation of the clinical relevance of chosen efficacy and harm outcomes is strongly recommended (addressed on page #9,10) |
| Participant timeline | 13 | Time schedule of enrolment, interventions (including any run-ins and washouts), assessments, and visits for participants. A schematic diagram is highly recommended (see Figure) (addressed on page #10,11, table1) |
| Sample size | 14 | Estimated number of participants needed to achieve study objectives and how it was determined, including clinical and statistical assumptions supporting any sample size calculations (addressed on page #12) |
| Recruitment | 15 | Strategies for achieving adequate participant enrolment to reach target sample size (addressed on page #12) |
| **Methods: Assignment of interventions (for controlled trials)** | | |
| Allocation: |  |  |
| Sequence generation | 16a | Method of generating the allocation sequence (eg, computer-generated random numbers), and list of any factors for stratification. To reduce predictability of a random sequence, details of any planned restriction (eg, blocking) should be provided in a separate document that is unavailable to those who enrol participants or assign interventions (addressed on page #8) |
| Allocation concealment mechanism | 16b | Mechanism of implementing the allocation sequence (eg, central telephone; sequentially numbered, opaque, sealed envelopes), describing any steps to conceal the sequence until interventions are assigned (addressed on page #8) |
| Implementation | 16c | Who will generate the allocation sequence, who will enrol participants, and who will assign participants to interventions (addressed on page #8) |
| Blinding (masking) | 17a | Who will be blinded after assignment to interventions (eg, trial participants, care providers, outcome assessors, data analysts), and how (addressed on page #8) |
|  | 17b | If blinded, circumstances under which unblinding is permissible, and procedure for revealing a participant’s allocated intervention during the trial (addressed on page #8) |
| **Methods: Data collection, management, and analysis** | | |
| Data collection methods | 18a | Plans for assessment and collection of outcome, baseline, and other trial data, including any related processes to promote data quality (eg, duplicate measurements, training of assessors) and a description of study instruments (eg, questionnaires, laboratory tests) along with their reliability and validity, if known. Reference to where data collection forms can be found, if not in the protocol (addressed on page #12) |
|  | 18b | Plans to promote participant retention and complete follow-up, including list of any outcome data to be collected for participants who discontinue or deviate from intervention protocols (addressed on page #12,7) |
| Data management | 19 | Plans for data entry, coding, security, and storage, including any related processes to promote data quality (eg, double data entry; range checks for data values). Reference to where details of data management procedures can be found, if not in the protocol (addressed on page #12) |
| Statistical methods | 20a | Statistical methods for analysing primary and secondary outcomes. Reference to where other details of the statistical analysis plan can be found, if not in the protocol (addressed on page #1) |
|  | 20b | Methods for any additional analyses (eg, subgroup and adjusted analyses) (NA. No subgroup analyses planned) |
|  | 20c | Definition of analysis population relating to protocol non-adherence (eg, as randomised analysis), and any statistical methods to handle missing data (eg, multiple imputation) (The statistical design does not forsee application of statistical methods to handle missing data. For this reason, a conservative termination/dropout rate was assumed for sample size calculations and the total number of patients increased accordingly. In case the case of a drop in the number of trial participants below a minimum number, the study protocol provides for the replacement of these participants by the recruitment and inclusion of additional study participants. To reduce the likelihood of missing data four measures will be implemented as addressed on page #12) |
| **Methods: Monitoring** | | |
| Data monitoring | 21a | Composition of data monitoring committee (DMC); summary of its role and reporting structure; statement of whether it is independent from the sponsor and competing interests; and reference to where further details about its charter can be found, if not in the protocol. Alternatively, an explanation of why a DMC is not needed (addressed on page #13) |
|  | 21b | Description of any interim analyses and stopping guidelines, including who will have access to these interim results and make the final decision to terminate the trial (NA, no interim analyses planned) |
| Harms | 22 | Plans for collecting, assessing, reporting, and managing solicited and spontaneously reported adverse events and other unintended effects of trial interventions or trial conduct (addressed on page #9) |
| Auditing | 23 | Frequency and procedures for auditing trial conduct, if any, and whether the process will be independent from investigators and the sponsor (addressed on page #16) |
| Ethics and dissemination | | |
| Research ethics approval | 24 | Plans for seeking research ethics committee/institutional review board (REC/IRB) approval (addressed on page #1) |
| Protocol amendments | 25 | Plans for communicating important protocol modifications (eg, changes to eligibility criteria, outcomes, analyses) to relevant parties (eg, investigators, REC/IRBs, trial participants, trial registries, journals, regulators) (addressed on page #6,7) |
| Consent or assent | 26a | Who will obtain informed consent or assent from potential trial participants or authorised surrogates, and how (see Item 32) (addressed on page #6,7, Only dedicated study physicians who have been authorized by the principal investigator and have been disclosed to the independent clinical data monitoring centre will recruit patients and gain informed consent. None of the physicians involved in the initial treatment i.e. implantation of a wireless SCS device, will be involved in recruiting patients and gaining informed consent. Patients will be approached after implantation of a wireless SCS device by study physicians who will explain the study in detail and, should the patient consider study participation, he or she will be handed a detailed information sheet which has been reviewed and approved by the local IRB.) |
|  | 26b | Additional consent provisions for collection and use of participant data and biological specimens in ancillary studies, if applicable (NA, no biological specimens will be collected in this study) |
| Confidentiality | 27 | How personal information about potential and enrolled participants will be collected, shared, and maintained in order to protect confidentiality before, during, and after the trial (addressed on page #6,7) |
| Declaration of interests | 28 | Financial and other competing interests for principal investigators for the overall trial and each study site (addressed on page #16) |
| Access to data | 29 | Statement of who will have access to the final trial dataset, and disclosure of contractual agreements that limit such access for investigators (addressed on page #16) |
| Ancillary and post-trial care | 30 | Provisions, if any, for ancillary and post-trial care, and for compensation to those who suffer harm from trial participation (NA, implantation of SCS device and handling of potential complications derived from SCS implantation are part of standard treatment procedures that are no affected by the trial) |
| Dissemination policy | 31a | Plans for investigators and sponsor to communicate trial results to participants, healthcare professionals, the public, and other relevant groups (eg, via publication, reporting in results databases, or other data sharing arrangements), including any publication restrictions (addressed on page #16) |
|  | 31b | Authorship eligibility guidelines and any intended use of professional writers (All authors have contributed to writing the present manuscript as detailed on page#17, no professional writers have been involved. After evaluation of the trial, results will be published in a peer-reviewed medical journal. Study physicians from participating study centres as well as other individuals involved in the study (e.g. data management, clinical data monitoring) can qualify as authors. |
|  | 31c | Plans, if any, for granting public access to the full protocol, participant-level dataset, and statistical code (The full study protocol and the statistical code are available from the corresponding author on reasonable request.) |
| Appendices |  |  |
| Informed consent materials | 32 | Model consent form and other related documentation given to participants and authorised surrogates (consent and documentation are addressed on page #6-7 and 15. The full consent and documentation forms are available from the corresponding author on reasonable request.) |
| Biological specimens | 33 | Plans for collection, laboratory evaluation, and storage of biological specimens for genetic or molecular analysis in the current trial and for future use in ancillary studies, if applicable (NA, no biological specimens will be collected in this study) |

*It is strongly recommended that this checklist be read in conjunction with the SPIRIT 2013 Explanation & Elaboration for important clarification on the items. Amendments to the protocol should be tracked and dated. The SPIRIT checklist is copyrighted by the SPIRIT Group under the Creative Commons “[Attribution-NonCommercial-NoDerivs 3.0 Unported](http://www.creativecommons.org/licenses/by-nc-nd/3.0/)” license.
